# XPO1-mutant NSCLC without STK11/KEAP1 mutations may predict better survival to immunotherapy

**DOI:** 10.1186/s12967-021-03098-5

**Published:** 2021-10-09

**Authors:** Xuanzong Li, Bing Zou, Shijiang Wang, Linlin Wang, Jinming Yu

**Affiliations:** 1 Department of Radiation Oncology, Affiliated Tumor Hospital of Xinjiang Medical University, Urumqi, China; 2 Department of Radiation Oncology, Shandong Cancer Hospital and Institute, Shandong First Medical University and Shandong Academy of Medical Sciences, Jinan, China

To the editor,

The nuclear export protein XPO1 regulates the export of a range of cargoes from the nucleus to the cytoplasm, and plays an important role in the maintenance of cellular homeostasis [1]. Previous studies suggested that XPO1 mutations participate in several steps of oncogenesis among multiple cancer types, and XPO1-mutant non-small-cell lung cancer (NSCLC) were associated with poorer survival contrast to their counterparts [2]. Therefore, finding novel strategies is urgently needed to improve the prognosis for XPO1-mutant NSCLC patients.

Up to now, the application of XPO1 blockade is challenging, and the definite efficacy for XPO1-inhibitors is still undetermined in NSCLC [1]. On the other hand, immune checkpoint inhibitors (ICIs)-based immunotherapies have changed the landscape of anti-cancer treatments, whereas the usage of immunotherapeutic agents should be careful in patients harboring driver mutations. Interestingly, Misako et al. demonstrated that NSCLC patients with XPO1 mutations were more likely to have high tumor mutational burden (TMB), which suggested that ICIs could serve as an option for these patients [2]. Nonetheless, to our best knowledge, there was no study reported the efficacy of ICIs in NSCLC patients with XPO1 mutations.

Using public databases, the prognostic values of several rare driver mutations were described in NSCLC patients with ICIs treatment. In this study, we integrated the MSK-TMB, POPLAR and OAK cohorts to evaluate the association between XPO1 mutation and the responses of NSCLC patients receiving ICIs [3, 4]. A total of 350 ICI-treated NSCLC patients from the MSK-TMB cohort and 429 atezolizumab-treated NSCLC patients with available genomic sequencing data (biomarker evaluable population) from the POPLAR and OAK cohorts were included in this study. Importantly, XPO1 mutations were defined as any nonsynonymous or fusion mutations. Our results showed that the incidence of XPO1 mutations was 1.2% (9 of 779) in the entire cohort. The clinical characteristics and the corresponding survival outcomes of the nine XPO1-mutant NSCLC patients were described in Table 1. In detail, most of patients were non-squamous (6 of 9, 66.7%), were male (7 of 9, 77.8%), had missense mutation type for XPO1 gene (7 of 9, 77.8%) and exhibited high TMB scores (above 10 mutations/Mb) (7 of 9, 77.8%). At first, we analyzed the overall survival (OS) in NSCLC patients with XPO1 mutations and their wild type counterparts. Unfortunately, we found patients harboring XPO1 mutations had comparable OS compared with those without the mutation (median OS, 19 months versus 12 months, p = 0.186; Fig. 1A). Notably, comutations of STK11/KEAP1 is common among NSCLC patients harbored XPO1 mutations [2], and NSCLC patients with STK11/KEAP1 mutations were associated with the inferior efficacy of ICIs which demonstrated both in MSK-TMB/POPLAR/OAK cohorts and other studies [5]. Hence, we further divided our patients into pure XPO1-mutant, wild type and XPO1-STK11/KEAP1 mutant groups. Surprisingly, NSCLC patients with pure XPO1 mutations had significantly longer OS than wild type and XPO1-STK11/KEAP1 mutant cohorts (median OS, not reached versus 12 months versus 4 months, p = 0.039; Fig. 1B).

**Table 1:** Clinical characteristics of XPO1 mutant NSCLC patients with immunotherapy

Patient	Study	Histology	Sex	XPO1 mutation (protein change)	Mutation type	TMB (mutations/Mb)	STK11/KEAP1 mutation	OS (months)	OS status
A	MSK-TMB	Squamous	Male	c.2461C > G (Q821E)	Missense	7.87	No	18	Living
B	MSK-TMB	Adenocarcinoma	Male	c.1087A > G (T363A)	Missense	22.63	No	21	Living
C	MSK-TMB	Adenocarcinoma	Male	c.1211C > G (P404R)	Missense	11.81	STK11	6	Dead
D	MSK-TMB	Adenocarcinoma	Female	c.1003C > G (Q335E)	Missense	10.82	No	13	Living
E	MSK-TMB	Squamous	Male	XPO1-USP34 fusion	Fusion	7.87	No	19	Dead
F	POPLAR	Squamous	Male	c.2977C > G (Q993E)	Missense	44	No	23	Living
G	POPLAR	Non-squamous	Female	c.1434G > C (E478D)	Missense	49	No	16	Dead
H	OAK	Non-squamous	Male	c.2461C > T (Q821*)	Nonsense	62	No	21	Living
I	OAK	Non-squamous	Male	c.1925G > T (G642V)	Missense	20	KEAP1	4	Dead

*NSCLC* non-small-cell lung cancer, *TMB* tumor mutational burden, *OS* overall survival

**Fig. 1:**
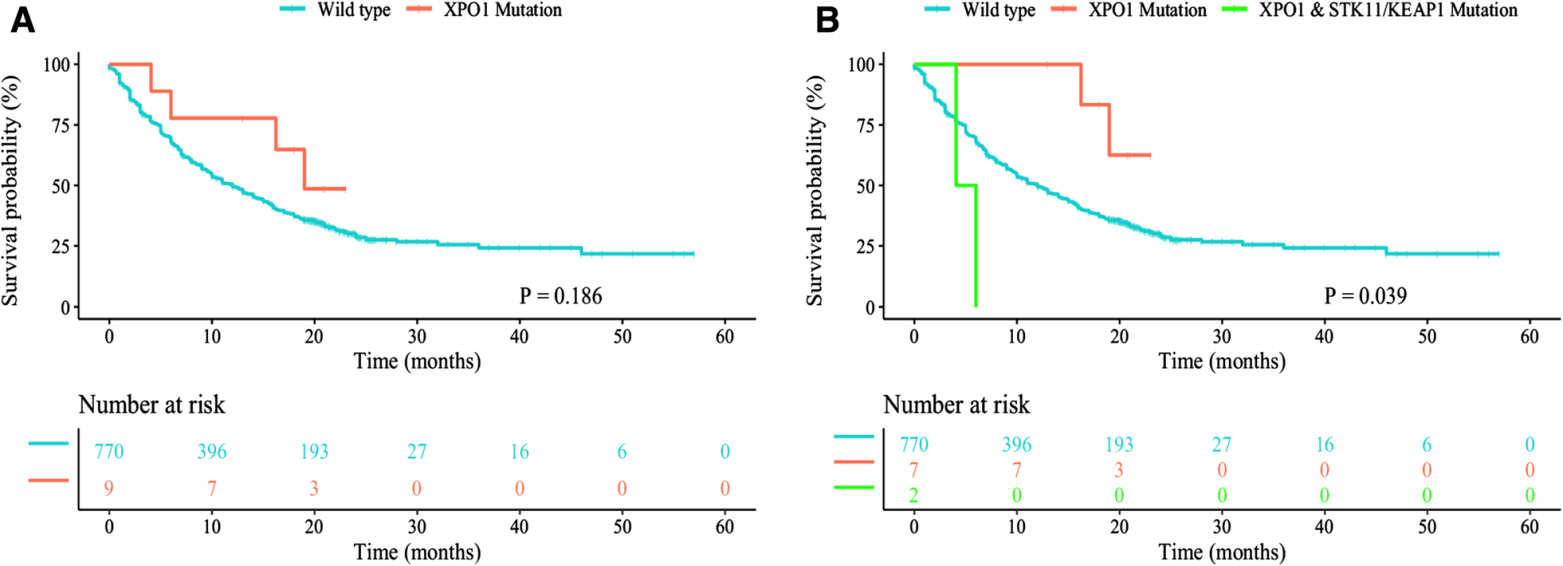
The prognostic value of XPO1 mutations in NSCLC patients with immune checkpoint inhibitors. **A** Kaplan–Meier curve comparing OS of NSCLC patients with XPO1 mutations and their wild type counterparts. **B** Kaplan–Meier curve comparing OS of NSCLC patients with pure XPO1 mutations, wild type and comutation of STK11/KEAP1 with XPO1 mutations. OS, overall survival; NSCLC, non-small-cell lung cancer

Collectively, for the first time, we investigated the role of XPO1 mutations in NSCLC patients with ICIs treatment. Our results suggested that there was no significant difference in the efficacy of ICIs therapy between NSCLC patients with and without XPO1 mutations. However, considering the co-mutation status of STK11/KEAP1, we demonstrated that patients with pure XPO1 mutations were correlated with longer survival and could serve as a favorable prognostic biomarker in NSCLC. Nevertheless, the small sample of XPO1-mutant NSCLC patients should not be neglected, and further studies will be necessary to verify our results.

## Data Availability

The data are available from the corresponding authors upon reasonable request.
